# Sociocultural Determinants of Risky Sexual Behaviors among Adult Latinas: A Longitudinal Study of a Community-Based Sample

**DOI:** 10.3390/ijerph13111164

**Published:** 2016-11-23

**Authors:** Patria Rojas, Hui Huang, Tan Li, Gira J. Ravelo, Mariana Sanchez, Christyl Dawson, Judith Brook, Mariano Kanamori, Mario De La Rosa

**Affiliations:** 1 Center for Research on U.S. Latino HIV/AIDS and Drug Abuse, Florida International University, Miami, FL 33199, USA; huanhu@fiu.edu (H.H.); tanli@fiu.edu (T.L.); gravelo@fiu.edu (G.J.R.); msanc062@fiu.edu (M.S.); cdawson@fiu.edu (C.D.); mkanamor@fiu.edu (M.K.); delarosa@fiu.edu (M.D.L.R.); 2 Department of Health Promotion and Disease Prevention, Robert Stempel College of Public Health & Social Work, Florida International University, Miami, FL 33199, USA; 3 Department of Biostatistics, School of Public Health, Robert Stempel College of Public Health & Social Work, Florida International University, Miami, FL 33199, USA; 4 School of Social Work, Robert Stempel College of Public Health & Social Work, Florida International University, Miami, FL 33199, USA; 5 New York University School of Medicine, New York University, 215 Lexington Avenue, New York, NY 10017, USA; Judith.Brook@nyumc.org

**Keywords:** risky sexual behavior, sociocultural determinants, mothers and daughters, HIV, Latinos, Hispanics, community, women, alcohol, drugs

## Abstract

Few studies have examined the sociocultural determinants of risky sexual behavior trajectories among adult Latinas. To longitudinally examine the link between sociocultural determinants of risky sexual behaviors, we followed a sample of adult Latina mother-daughter dyads (*n* = 267) across a 10-year span through four waves of data collection. The present study investigates how risky sexual behavior (operationalized as sex under the influence of alcohol or other drugs, sex without a condom, or multiple sex partners) is affected by: (a) socioeconomic conditions; (b) mental health; (c) medical health; (d) acculturation to U.S. culture; (e) interpersonal support; (f) relationship stress; (g) mother-daughter attachment; (h) intimate partner violence; (i) religious involvement; and (j) criminal justice involvement. Results indicate the following factors are negatively associated with risky sexual behavior: drug and alcohol use, treating a physical problem with prescription drugs, religious involvement, and mother–daughter attachment. The following factors are positively associated with risky sexual behavior: higher number of mental health symptoms, being U.S.-born, and criminal justice involvement. We discuss implications for the future development of culturally relevant interventions based on the study findings.

## 1. Introduction

HIV poses a serious threat to Latino women aged 35 to 54 [1]. In 2013, Latinos accounted for 23% or 10,888 of the estimated 48,145 cases of HIV infection in the United States [1]. Of the HIV diagnoses among Latinos, nearly 15% or 1610 were Latinas—86% of whom contracted the virus through heterosexual contact [1]. In 2014, among ethnic/racial groups of women, Latinas accounted for an estimated 15% of new HIV infections and their HIV incidence rate was nearly four times the rate of non-Latina white women [2]. Furthermore, in terms of HIV prevalence, among women living with an HIV diagnosis in 2010, 19% were Latina whereas 18% were non-Latina white women [2]. To better understand the reasons for the disproportionately high rates of HIV infection among Latinas, the present study examines the sociocultural determinants of risky sexual behavior of Latinas in the United States.

In recent decades, research studies have investigated risky sexual behaviors among Latinos; most have focused on adolescent Latinas [3,4,5], while few have focused on adult Latinas. Furthermore, the studies on adult Latinas have been predominately cross-sectional [6,7]. Therefore, in terms of intervention development, they offered only limited information on the predictors of risky sexual behavior. The present study uses data from one of the few existing longitudinal investigations on adult Latinas in South Florida—documenting the association between sociocultural factors and HIV risk behaviors over time. Here, risky sexual behavior is defined as engaging in any of the following behaviors in the past 12 months: (a) sex under the influence of alcohol or other drugs; (b) vaginal or anal sex without a condom; or (c) more than one sex partner. Among Latinas, we hypothesize that the following will lead to increases in risky sexual behaviors over time: lower levels of socioeconomic status, social support, and mother-daughter attachment; and higher levels of substance misuse, acculturation to the United States, social stress, neighborhood stress, poor mental health, and intimate partner violence. We discuss implications for the future development of culturally relevant interventions based on study findings.

### 1.1. Social and Cultural Determinants of Health and Latina Risky Sexual Behaviors

#### 1.1.1. Individual Determinants

Past studies have reported findings on individual determinants (e.g., age, socioeconomic conditions, and marital status) related to risky sexual behaviors among Latinas. Married Latinas and older Latinas are more likely to engage in risky sexual behavior, specifically unprotected vaginal sex [8]. Moreover, Latinas living with a partner and and/or children—and those who are employed—have higher levels of unprotected vaginal sex [9] and Latinas with lower educational levels and higher income are more likely to have low levels of condom use [10]. Latinas diagnosed with a mental health disorder are more likely to engage in risky sexual behavior; by examining data collected across several time points, the present study sheds light on the effect of time on risky sexual behavior [11]. The present study also allows us to determine the effect of age and risky sexual behavior—answering the question of whether being a mother, regardless of age, is significantly correlated with lower HIV risky sexual behaviors. Additionally, the present study addresses the gap in the literature pertinent to adult Latinas having sex under the influence of drugs or alcohol. Worthy of note: recent Latina immigrants have been shown to exhibit lower levels of risky sexual behavior compared to their U.S.-born counterparts [12].

#### 1.1.2. Cultural Environment Determinants

The impact of cultural factors on the risky sexual behavior of Latinas has been well documented. Studies have consistently found that as Latinas acculturate, they are exposed to U.S. norms that are more accepting of female premarital sexual behavior—making Latinas more likely to engage in risky sexual behavior, thereby increasing their risk of contracting HIV [8,12,13]. Lee and Hahm (2010) found that more acculturated Latina adolescents who spoke English at home were more likely to engage in risky sexual behaviors than Latina adolescents who were foreign-born and did not speak English at home [14]. 

However, traditional Latino gender roles—*marianismo* and *machismo*—also heighten Latinas’ risk of HIV infection [15,16,17,18]: according to machismo, males should be sexually dominant and have multiple sex partners; according to marianismo, Latina women should be docile and obedient [17]. Relatedly, Latinas who have less control in the relationship are significantly more likely to have unprotected sex with their primary partners [19]. These gender roles influence issues surrounding sexual behavior, including condom use, partner communication, and finding sexual partners [20]. 

The need for longitudinal studies examining sociocultural factors associated with HIV risk behaviors is evident in the literature. The current study presents statistically significant results irrelevant of time and in the case of the mother and daughter dyads, we will be able to disentangle the effect of age from the status of being a mother. Furthermore, we could see how social support, particularly belonging and tangible support, decreased over time, and most noticeably during the first five-year follow-up. Equally significant was the decrease of alcohol misuse, which indicates that Latina may be consuming alcohol with family members (who provided social support) and within their relationships. The longitudinal study will not prove a causal effect but it will demonstrate that a high correlation of the variables is irrespective of time. 

#### 1.1.3. Interpersonal Support and Attachment 

Social support is critical in the lives of Latinas, especially given that these women often hold traditional Latino values such as *personalismo* (a cultural value that involves building interpersonal relationships and the development of friendly and warm personal connections), *familismo* (a cultural value that involves a sense of family connection and attachment, sense of loyalty and solidarity to the nuclear and extended family members), and *confianza* (a cultural value that involves confiding in others) [21,22]. Latinas who receive tangible and emotional support, and have strong attachments to their families, cope better with stress and report less alcohol and illicit drug use compared to Latinas without support—lowering their risk of engaging in risky sexual behavior [4,7,23,24,25,26]. After controlling for age, sex, and homelessness among Puerto Rican women, Mino et al. (2006) found that emotional support was negatively associated with drug injection frequency (standardized coefficient = −0.168, *p* = 0.02), reducing the risk of injection-related HIV infection [27]. Additionally, interculturality and social support have been found to be associated with lower risk of drug injection and risky sexual behaviors [28]. In a cross-sectional study, attachment was found to be associated with sexual risk among Latinas, with higher levels of attachment between mothers and daughters being protective against the use of drugs before sex [29]. However, Latinas who receive social support from partners are more likely to engage in unprotected vaginal sex [9]. We hypothesize thatsocial support will be associated with having sex without condoms among Latinas who engage in sex with trusted primary partners.

#### 1.1.4. Intimate Partner Violence

Among Latinas, the relationship with their intimate partner is essential to their sexual health and well-being. Yet, Latinas are disproportionately impacted by intimate partner violence (IPV) [30]. Cavanaugh et al. (2014) found the prevalence of sexual partner violence (violence where sexual abuse is involved) to be 38% among a large sample of Latina women. IPV has been found to be significantly associated with place of birth: among physically abused Latinas, foreign-born Latinas have 2.10 times the odds (95% CI: 1.44–3.07) of experiencing sexual partner violence compared to U.S.-born Latinas [31]. Conversely, Moreno et al. (2011) found that those who were born on the U.S. mainland and reported English language preference reported more IPV than those who were born in Puerto Rico [32]. IPV is linked to substance use behaviors and other risky health behaviors and sociocultural factors among poor Latinas living in an urban setting, thereby increasing their risk of engaging in risky sexual behaviors [17,33,34,35,36]. IPV increases the risk of HIV among Latinas due to an increased likelihood of engaging in risky sexual behavior [16,37,38]. Specifically, women experiencing IPV are at increased HIV risk because many of them are forced to have sex with their infected partners and they have a limited or compromised ability to negotiate safe sex practices such as condom use [17,38]. Randolph et al. (2011) found that sexual abuse was correlated with lower condom use and negotiation power, while extensive IPV had the strongest association with higher HIV/STI risk after controlling for relationship status, sexual abuse, and relationship power [37].

#### 1.1.5. Chronic Stress 

Chronic stress is a significant factor associated with increased risky sexual behavior among Latinas. Vega et al. (1998) found that the lifetime prevalence for any anxiety disorder was 18% among Mexican American immigrant women and 27% among Mexican Americans born in the United States—levels that were higher than those of their male counterparts [39]. Increased stress leads to increased rates of alcohol and/or substance use among Latinas [40]. Utilizing a large sample of female Spanish speaking youths, Teva et al. (2010) reported that social stress increased the likelihood of drug use at last sexual encounter among females [41]. For Latinas, much of their stress can be directly attributed to their daily life and social environment [39,42,43,44,45], which often include poor socioeconomic conditions, experiences of discrimination, undocumented immigration status, lack of access to social/medical services, limited educational opportunities due to cultural and language barriers [46,47,48,49], and a lack of access to disease prevention education, health promotion intervention programs, and health insurance [50]. 

#### 1.1.6. Drug and Alcohol Use 

Female drug users, in general, are more likely to engage in risky sexual behaviors compared to their peers who do not use drugs [51]. In a previous study examining recent Latino immigrants’ alcohol abuse and risky sexual behaviors, Rojas et al. (2014) found that alcohol was associated with lower condom use frequency, sex under the influence, and having more sexual partners [52]. Young adult Latinas who frequently use alcohol [53] or illicit drugs [54] are more likely to engage in unprotected sex compared to those who used alcohol less frequently or not at all. However, most existing research in this area has examined adolescents, and it is unclear whether the association persists in adult women. Moreover, the association of alcohol/drug use and risky sexual behavior among Latinas has received even less attention in the research literature.

Preliminary studies linking drug and alcohol use to unsafe sex suggest that substance-using Latinas are more likely to engage in unsafe sexual practices; however, these studies often do not focus on sexual behaviors that occur while the woman is intoxicated. Such behaviors are especially risky due to the effects of drugs and alcohol on cognition and judgment in substance users [55]. Intoxication increases the likelihood of impulsive and risky decisions and decreases inhibition [56], which may, in turn, increase the likelihood of unsafe sex. De La Rosa et al. (2010) found that single and older Latinas, and daughters with drug-using mothers, were more likely to engage in unprotected sex under the influence of alcohol and other drugs [29]; as drug use decreases with time, we expect to find decreases in sex under the influence in the present sample.

#### 1.1.7. Community Environment Determinants 

During the last decade, researchers have paid more attention to the influence of community environment on risky sexual behaviors; however, studies focusing on the effects of neighborhood on the risky sexual behavior of Latinas remain scarce. Kulis et al. (2007) found that Latinas living in neighborhoods with fewer institutional supports and higher levels of crime have poorer health outcomes [57]. Moreover, neighborhood organizational resources (e.g., churches) are associated with decreased risky sexual behaviors, while neighborhood stress (e.g., crime) is associated with increased substance misuse [58,59,60,61,62]. Researchers posit that residents exposed to neighborhood crime are more likely to perceive themselves as potential victims of crime and, in turn, are more likely to use substances to cope with stress—which then increases their likelihood of engaging in risky sexual behavior. Additionally, Latinas involved in gangs are exposed to more community violence and limited access to reproductive health care services, along with more sexual coercion from their male partners, thereby increasing their involvement in risky sexual behavior [63]. Research on this topic in other populations has been extensive. A study on neighborhood collective efficacy and risky sexual behavior among youth living in urban settings reported that collective efficacy is negatively associated with having two or more sexual partners—although this effect was dependent on age [64]. Moreover, Parrado and Flippen (2010), assessing the impact of neighborhood violence stress on sex worker use among Hispanic migrants, found that lower levels of collective efficacy—as measured by social isolation—and increased levels of gender imbalance are both positively associated with sex worker use [65]. 

#### 1.1.8. Religious Involvement 

Religion plays an important role in Latino culture [66,67]. Yet, with two notable exceptions [24,68], there continues to be a lack of research on the influence of religious involvement on the risky sexual behavior of adult Latinos in general—and even less research has focused on adult substance-using Latina women [48,68,69]. Using a sample of 415 recent Latino immigrants, Sanchez et al. (2015) reported that positive (adaptive) religious coping (e.g., seeking help from God in letting go of anger) were associated with lower levels of alcohol misuse, while negative (maladaptive) religious coping (e.g., wondering what I did for God to punish me) led to higher levels of alcohol misuse in study participants [24]. In another study, Smith (2015) found that religious involvement/participation was protective against risky sexual behavior among adult Latinas, while non-participation increased the risk of engaging in risky sexual behavior [70]. Additionally, examining trends from 1995 to 2008 of religion and risky sexual behaviors among Latina adolescents, Edwards et al. (2011) found that Latinas who reported that religion was very important to them, and Latinas who frequently attended religious services, were less likely to have had sex, had fewer partners, and were older at sexual debut [71]. However, the intense identification with religion may promote traditional gender roles in Latino culture, placing Latinas at greater risk of engaging in risky sexual behavior (e.g., reduced condom use) and contracting HIV [72]. 

#### 1.1.9. Involvement with the Criminal Justice System 

Involvement in the criminal justice system leads to higher levels of risky sexual behavior and subsequently HIV/STI levels [6,73,74]. The present study addresses the gap in the literature on the complexity of sociocultural determinants of health; it addresses two questions: Is acculturation a protective factor for HIV risk behaviors among adult Latina women? And, is involvement in criminal activities a risk factor for HIV infection? Prevalence of HIV has been found to be higher among female inmates (3%) than male inmates (2.5%) [75]. Inmates are at higher risk of having sexual intercourse at an early age, unprotected sex, multiple sex partners, lax attitudes toward sex, and low self-efficacy in regard to safe sexual practices [74]. In particular, adolescent females are at increased risk for HIV infection, along with drug addiction, mental illness, poor physical health, and domestic violence [76]. Women involved in the juvenile justice system have been found to be sexually active, have inadequate information on sex, and used little or no birth control or any other reliable contraceptive method in the past month [77]. Research on Latinas in the criminal justice system is scarce, although studies have examined the system’s effects on risky sexual behavior in other populations, predominately youth. Knittel et al. (2013) found that among Latino males, incarceration is associated with an increased rate of lifetime sexual partnership, although this was attenuated by substance use. Criminal justice involvement has also been associated with increased odds of having multiple sexual partners before and after incarceration [73]. Belenko et al. (2004) found that individuals on probation and parole have high rates of unprotected sex, and limited exposure to effective HIV education and prevention interventions, thereby increasing their risk of contracting HIV [78]. Past trauma predicts criminal activity, thus influencing these individuals’ risk of engaging in risky sexual behavior and contracting HIV [79]. 

### 1.2. Conceptual Framework

Our study was guided by Bogenschneider’s (1996) ecological risk/protective model and a growing body of work on social and cultural influences on health (e.g., [5,80,81]). This conceptual model integrates proximate causes of disease, including individual factors (e.g., substance use), and distant causes of disease, including social factors (e.g., social support). This socioecological theory emphasizes multiple contextual influences as occurring at different levels, including family and community. These levels also exert influences on the health behaviors of adult women. In adult Latino women, factors may include level of social support, availability of resources, and community norms. As indicated in Bogenschneider’s theory, the conceptual framework of the ecological model is marked by the importance of family and relationships in the life of the individual. Grounded in the importance of family (familism) and relationships for Latinas, our study is guided by this theoretical model. The socioecological perspectives suggest that at the micro-level, family relationships are a primary context for human behavior development. Relationships with family members, particularly mothers and daughters, in Latinos play a major role in shaping behavioral patterns. Thus, the conceptual framework in the present study accounts for socialcultural determinants in five domains: individual, cultural, interpersonal, community, and institutional. The present study addresses the following question: Will lower levels of socioeconomic status, social support, and mother-daughter status—along with other sociocultural factors—affect HIV risky sexual behaviors over time? 

## 2. Materials and Methods

Data for the present study were obtained from a longitudinal study of intergenerational drug use and related health behaviors among Latina mother-daughter dyads in Miami-Dade County, Florida. The study was approved by, and conducted in compliance with, the institutional review board at Florida International University, a public university in the southeastern United States (IRB-13-0305).

### 2.1. Procedures

We collected four waves of data. At baseline (Wave 1), a community-based, convenience sample (*n* = 316) of substance using and non-using participants was recruited via the snowball sampling (chain referral) method [82]. Data was collected during the years 2006 (baseline) and between 2011 and 2013 (three consecutive, yearly follow-ups). The five-year gap was due to funding lapses. The sample was designed to include similar numbers of drug-using and non-using mothers and daughters; participants were recruited by posting flyers at various venues: community health fairs, health clinics, drug court programs, Alcoholics Anonymous and Narcotics Anonymous meeting sites, Spanish radio stations and television channels, and a local newspaper. Inclusion criteria did not require dyads to be uniformly drug users or non-users, and in drug-using dyads the inclusion criteria did not require dyad members to use the same drug(s). Study methods and sample selection are described in detail elsewhere [29]. At baseline, the average age was 52.51 (S.D. = 10.36) among mothers, and 27.35 (S.D. = 9.18) among daughters. Forty-three percent of daughters (*n* = 57) and 13% (*n* = 17) of mothers were U.S.-born. 

All consenting study participants completed a face-to-face interview guided by a structured questionnaire that lasted approximately 1.5 h, and participants received incentives of $40.00 at baseline and $50.00 at each follow-up. Consents and interviews were performed in English and Spanish, and women were interviewed in the language, and at the location, of their preference. Trained female bilingual interviewers collected the data—which mostly occurred in participants’ homes. The first follow-up (Wave 2) was conducted five years after the baseline (Wave 1) interview; data collection in Wave 3 and Wave 4 occurred six6 and seven years, respectively, after baseline. Of the 316 baseline participants, Wave 2 included interviews with 295 (93%) participants, Wave 3 included interviews with 292 (92%), and Wave 4 included interviews with 278 (88%). However, the present study sample includes 272 participants who participated in all four waves and were not missing data points, including dyads that may have lost one member. For women who were lost to follow-up, the primary reasons were death and moving to another country. There are no significant differences between those who participated in fewer than four waves of data collection and those who participated in all four waves of data collection or were lost to follow-up.

#### 2.1.1. Measures

**Demographics**. A demographics form assessed, in part, participants’ age, marital status, number of years living in the United States, education, and employment status.

**Medical and mental health status**. We used the Addiction Severity Index [83] to assess medical and mental health status. For medical health, participants answered two questions: (1) Do you have any chronic medical problems that continue to interfere with your life? and (2) Are you taking any prescribed medication on a regular basis for a physical problem? Regarding mental health, participants reported their experience of seven mental disorder symptoms: depression; anxiety; hallucination; difficulty understanding, concentrating, or remembering difficulty controlling violent behavior including episodes of rage or violence; serious thoughts of suicide; and suicide attempts. We use the total count of mental disorder symptoms and depression in the analysis. 

**Acculturation**. We use two variables as a proxy for acculturation: country of birth (U.S.-born vs. foreign-born) and level of English-speaking proficiency. Participants responded on a four-point Likert-type scale ranging from low to high: 1 = *not at all* to 4 = *excellent*. 

**Social support**. We use the Interpersonal Support Evaluation List [84] to measure two distinct dimensions of family and friends’ social support: Tangible (i.e., the perceived availability of material aid when needed) and Belonging Support (i.e., the perceived availability of people with whom they can do activities). The scale indicated good reliability estimates at baseline: α = 0.86 for mothers and α = 0.88 for daughters for the Tangible Support scale; α = 0.78 for mothers and α = 0.74 for daughters on the Belonging Support scale. 

**Attachment**. We use the Inventory of Parent and Peer Attachment (IPPA) [85] to assess attachment. IPPA contains 25 items in three dimensions: trust, communication, and alienation. Each item is answered on a five-point Likert-type scale ranging from 1 = *almost never or never true* to 5 = *almost always or always true*. We use a total score in our analyses. The scale indicated reliability estimates for the total sample at baseline (α = 0.93) and for mothers and daughters separately (α = 0.93 for daughters; α = 0.92 for mothers). 

**Intimate partner violence**. We used the Addiction Severity Index to assess whether the participant had experienced emotional, physical, and/or sexual abuse. If a participant reported their sexual partner or spouse as the perpetrator, we coded the person as 1 for the variable intimate partner violence; all others were coded as 0.

**Multiple sex partners**. Using the same measure adapted from Turner’s Transition study, which examined the substance use and risky sexual behavior trajectories of young adults [86], we asked participants how many sexual partners they had in the last 12 months. For the dependent variable of having multiple sex partners, participants who reported more than one partner were coded as 1, while those who reported no more than one partner were coded as 0. 

**Sex under the influence**. Using the same measure adapted from Turner’s Transition study, we asked participants if, in the last 12 months, they or their partner drank alcohol or used any other drug before or during sex. Participants responded on a five-point Likert-type scale ranging from low to high: 1 = *Always*, 2 = *Usually*, 3 = *Sometimes*, 4 = *Rarely*, and 5 = *Never*. For the dependent variable sex under the influence, the participants who reported between 1 = *Always* and 4 = *Rarely* were coded as 1, while those who reported 5 = *Never* were coded as 0. 

**Sex without a condom**. We measured condom use with an adapted and shortened measure of sexual behavior from Turner’s Transition study. We asked participants about their sexual behavior in the past 12 months: “How many times did you have vaginal sex?”, “How many times did you have oral sex?”, and “How many times did you have anal sex?” To assist participant recollection, they were encouraged to think about how many times a week they had sex. If they reported once or more, participants were asked to report the number of times they or their partners used condoms (“How many of those times did you or your partner use condoms?”). For the dependent variable sex without a condom, participants who reported using condoms as often as they had sex were coded as 0, while participants who used condoms less often than the number of times they had sex were coded as 1. 

**HIV risk sexual behaviors**. For the dependent variable HIV risk sexual behaviors, participants’ reports of three dependent variables are combined: sex without condom, multiple sex partners, and sex under the influence. Participants who reported multiple sex partners, reported using condoms sometimes or never, and reported sex under the influence were coded as 1, while those who reported none of these behaviors were coded as 0. 

**Chronic stress**. We assess stress using a previously validated measure of adult health risk behaviors [87]. The measure includes four Chronic Stress subscales: Employment, Relationships, Social Life & Recreation, and Neighborhood. In our analysis, we use only three subscales (omitting the employment subscale), as at each time point, approximately half of the sample reported being unemployed. At baseline: for the Relationships subscale, Cronbach’s alphas were 0.87 (total sample), 0.87 (daughters), and 0.87 (mothers); for the Social Life & Recreation subscale, Cronbach’s alphas were 0.49 (total sample), 0.47 (daughters), and 0.52 (mothers); and for the Neighborhood subscale, Cronbach’s alphas were 0.70 (total sample), 0.71 (daughters), and 0.69 (mothers).

**Religious involvement**. We use the Santa Clara Strength of Religious Faith Questionnaire [88] to measure religious involvement. We use participants’ average response to two statements, including “I pray daily” and “I consider myself active in my faith”. The assessment uses a four-point Likert-type scale ranging from 1 = *strongly disagree* to 4 = *strongly agree*. 

**Criminal justice system involvement**. Using one item from the Addiction Severity Index, we assess criminal justice involvement. If a participant answered “Yes” to the question “Have you been involved in any criminal activity in the last 12 months? (Probe: Include offenses such as major driving violations.)”, the person is coded as 1 for criminal justice involvement. 

**Alcohol misuse**. We use the Health and Daily Living Form [89] to assess alcohol misuse. A participant was considered to misuse alcohol if she reported at least one of the following drinking episodes on at least one occasion once per month: (a) 4–5 glasses of wine or more; (b) 3–4 cans/bottles of beer or more; (c) 3–4 drinks or more with 4 oz. of alcohol in each drink; (d) 5–7 drinks or more with 2 oz. of alcohol in each drink. 

**Drug misuse**. We use the Drug Use Frequency measure [90] to assess drug misuse. A participant was considered to misuse drugs if she reported at least one of the following: (a) marijuana use at least three days per week; (b) cocaine use at least two times per week; (c) heroin use at least one time per week; (d) ecstasy use at least three times per month; (e) using prescription medications (e.g., sedatives, tranquilizers, sleeping pills, antidepressants) for a longer time than prescribed, in greater amounts than prescribed, or without doctor’s authorization or prescription during the past 12 months. The variable was coded dichotomously with any of the abovementioned behaviors coded as 1 = drug misuse; all others were coded as 0 = no drug misuse.

#### 2.1.2. Analysis

Descriptive statistics characterize the sample at each of the four waves. Summary statistics are compiled for all variables—proportions for categorical variables and means and standard deviations for continuous variables. 

Hierarchical modeling is used to examine effects of sociocultural determinants on HIV risk behaviors over time. We use PROC GLIMMIX in SAS/STAT 9.3 (User’s Guide, 2011, SAS Institute Inc., Cary, NC, USA [91]) as it allows for modeling data with a nested structure. We decided to use hierarchical modeling based on our data structure, which comprises two levels of nested structure: first, four waves of data are nested within the same individual (i.e., mother or daughter); second, mother and daughter data are nested within the family. Initially, we hypothesized that each individual has a different rate of change over time, and tested an unconditional individual growth curve model. To do so, we included both intercept and time in the random statement in PROC GLIMMIX. However, the unconditional model shows that the estimate of variance of the time slope was not significantly different from 0. Therefore, we do not include time in the random statement in the final reported models. However, we still model the two levels of nested structure, and include time as an independent variable. Specifically, we use individuals’ reports of number of years in the United States at each wave as the time variable, as an increased number of years in the United States was consistent with time gaps between data collection waves.

We modeled four binary variables: HIV risk behaviors, multiple sex partners, sex under the influence, and sex without a condom. Exponential values of all results are reported in the tables. An exponential value less than 1 indicates decreased HIV risk behaviors, while greater than 1 indicates increased HIV risk behaviors. The specific data analysis steps were: Step 1, we added each of the time-varying sociocultural determinants to the model; and Step 2, we simultaneously included all of the time-varying sociocultural determinants of statistical significance in Step 1. Given the nested structure of data, in all models we included three variables: number of years in the United States at each wave, age of arrival in the United States, and generational status (i.e., mother or daughter).

## 3. Results

### 3.1. Descriptive Analysis

As presented in Table 1, the overall HIV risk behaviors and each of the three HIV risk behaviors (i.e., multiple sex partners, sex under the influence, and sex without condoms) decrease over time. Similarly, the rate of alcohol misuse also decreases over time from 31% at baseline to 19% at Wave 4. However, the rate of other drug misuse (e.g., sedatives, marijuana) is stable over time. There is no significant change over time regarding the English speaking skills of the participants, which is not surprising given that Miami-Dade County is a largely Spanish speaking community where English language ability is not necessary [92]. A small percentage of participants reported depression symptoms, showing a significant difference of 11 percentage points between baseline and Wave 2, and continuing to decrease over the last two waves (19% at the Wave 3 and 9% at the Wave 4). As expected, the mean of mother-daughter attachment score increases slightly over time. 

### 3.2. Bivariate Analysis 

We present results from bivariate analyses in Table 2. As stated above, the analysis was done by adding each of the time-varying sociocultural determinants to the model.

**Overall HIV risk behaviors**: being the mother within the dyad; age at baseline; religious involvement; being married, remarried, or with a partner; and being widowed, separated, or divorced (compared with never married) have significant negative associations with overall HIV risk behaviors. The following factors are associated with more risky sexual behaviors: higher number of mental health symptoms reported, being U.S.-born, English proficiency, tangible support, belonging support, intimate partner violence, criminal justice system involvement, alcohol misuse, and drug misuse. 

**Multiple sex partners**: being the mother within the dyad; age at baseline; religious involvement; mother-daughter attachment; being married, remarried, or with a partner; and being widowed, separated, or divorced (compared with never married) have significant negative associations with the risk of having multiple sex partners. The following factors are associated with higher risk of having multiple sex partners: higher number of mental health symptoms reported, being U.S.-born, English proficiency, belonging support, intimate partner violence, criminal justice system involvement, alcohol misuse, and drug misuse. 

**Sex under the influence**: being the mother within the dyad; age at baseline; religious involvement; mother-daughter attachment; being married, remarried, or with a partner; and being widowed, separated, or divorced (compared with never married) have significant negative associations with the risk of sex under the influence. The following factors are associated with higher risk of sex under the influence: self-report of depression, higher number of mental health symptoms reported, being U.S.-born, English proficiency, tangible support, belonging support, intimate partner violence, criminal justice system involvement, alcohol misuse, and drug misuse. 

**Sex without a condom**: being the mother within the dyad; age at baseline; religious involvement; mother-daughter attachment; being married, remarried, or with a partner; and being widowed, separated, or divorced (compared with never married) have significant negative associations with the risk of sex without a condom. The following factors are associated with higher risk of sex without condom: higher number of mental health symptoms reported, being U.S.-born, English proficiency, intimate partner violence, criminal justice system involvement, alcohol misuse, and drug misuse. 

### 3.3. Multivariate Analysis 

We present results from multivariate analyses in Table 3. As stated above, the analysis was done by simultaneously including all of the time-varying sociocultural determinants of statistical significance in the bivariate analyses. 

**Overall HIV risk behaviors**: in the multivariate analysis, age was the only variable at baseline (Exp. (β) = 0.93, *p* < 0.001) that was negatively associated with overall HIV risk behavior, while intimate partner violence (Exp. (β) = 1.88, *p* < 0.05); criminal justice system involvement (Exp. (β) = 6.06, *p* < 0.05); being married, remarried, or with a partner (compared with never married) (Exp. (β) = 7.06, *p* < 0.001); and drug misuse (Exp. (β) = 5.09, *p* < 0.001) were positively associated with overall HIV risk behaviors. 

**Multiple sex partners**: similarly, the results show that age at baseline (Exp. (β) = 0.90, *p* < 0.001) and being married, remarried, or with a partner (compared with never married) (Exp. (β) = 0.27, *p* < 0.001) were negatively associated with the risk of having multiple sex partners, while only drug misuse (Exp. (β) = 2.11, *p* < 0.05) was positively associated with the risk of having multiple sex partners. 

**Sex under the influence**: results show that at baseline, the only variable that was (Exp. (β) = 0.95, *p* < 0.01) negatively associated with sex under the influence was age; while criminal justice system involvement (Exp. (β) = 6.43, *p* < 0.001), alcohol misuse (Exp. (β) = 2.64, *p* < 0.001), and drug misuse (Exp. (β) = 2.40, *p* < 0.01) were positively associated with sex under the influence. 

**Sex without a condom**: similar to sex under the influence, only age at baseline (Exp. (β) = 0.96, *p* < 0.01) was negatively associated with sex without a condom, while intimate partner violence (Exp. (β) = 2.51, *p* < 0.001); criminal justice system involvement (Exp. (β) = 3.01, *p* < 0.05); being married, remarried, or with a partner (compared with never married) (Exp. (β) = 7.47, *p* < 0.001); and drug misuse (Exp. (β) = 3.53, *p* < 0.01) were positively associated with sex without a condom.

## 4. Discussion

The present study longitudinally examines the sociocultural determinants of HIV risk sexual behaviors among adult Latina mother-daughter dyads. Results indicate significant associations between various social-cultural determinants and the HIV risk behaviors in the present sample. First, our findings suggest that among adult Latinas, IPV may have a significant long-term influence on risky sexual behaviors in general, and may specifically impact engagement in unprotected sex. These results are congruent with the literature and highlight that IPV is a significant and prevalent and significant public health risk factor associated with the increase of HIV infection among Latina women. 

Second, we found religious involvement to be a protective factor in the bivariate analysis, as it is negatively associated with having multiple sex partners and sex under the influence. These results support findings from previous research indicating that religious involvement is central to Latinas and Latino culture, and that religious components should be incorporated into interventions targeting adult Latinas [68]. Unexpectedly, religious involvement was neither a protective nor risk factor for sex without a condom in the multivariate analysis. It may be that religious involvement was confounded by other factors such as marital status (being married or having a main partner) and the unlikelihood that Latino women will negotiate condom use with their main partners. Other factors that are potentially confounding our results are: beliefs that Latinas should be modest and sexually naïve, and cultural normative expectations for Latinas not to discuss condom use with main partners [19]. Lastly, demographic factors such as age, as various studies have shown, increase religious involvement; additionally, as they age, women are less likely to need to use condoms as they may be using other methods of pregnancy prevention and may be having sex only with their main partner [93].

Third, involvement in the criminal justice system is predictive of various HIV risk behaviors including the overall HIV risk behaviors, sex under the influence, and sex without condoms. These results parallel previous study findings [94] and suggest that HIV prevention and intervention programs need to target women who are either at risk of becoming involved, or already involved with the criminal justice system. Our results suggest that Latinas may benefit from collaborations between the criminal justice system and community-based public health prevention programs targeting HIV risk in adult Latinas. For instance, driving under the influence offenders must attend mandatory classes, which could provide the Department of Health with an avenue to educate female offenders about healthy decision-making, especially health decisions pertaining to sexual behaviors. This effort would be particularly timely and necessary, given that Latinas represent the largest racial/ethnic group among female offenders [95]. Although, Latinos/as are less likely to seek and participate in treatment than individuals from other ethnic groups [96], the large number of Latinos/as involved with the criminal justice represents a significant public health concern. Institutionalizing prevention interventions and health promotion programs within correctional facilities and drug court-mandated programs could help these Latinas decrease their risks. 

Fourth, compared with never married, being married, remarried, or with a partner is associated with multiple HIV risk behaviors—though the associations are in different directions. Specifically, being married, remarried, or with a partner is positively associated with the combined HIV risk behaviors among Latinas and sex without a condom, while it is negatively associated with the risk of having multiple sex partners. These sexual risks are expected as Latinas’ sexual behaviors are influenced by gender roles and cultural values such as marianismo and they are less likely to discuss issues related to sexual behaviors and use condoms with their main partners [20]. First, these results suggest that HIV prevention must target young women and their committed partners—teaching them condom use and safer sex negotiation skills. Second, due to the high prevalence of monogamy (not reporting multiple sex partners) among participants, which functioned as a sociocultural protective factor, public health practitioners should consider encouraging this behavior in future HIV prevention intervention and among younger Latinas. 

Fifth, our findings indicate that being U.S.-born is not associated with combined risky sexual behaviors (having sex without condoms, multiple sex partners, and sex under the influence of alcohol). The present study’s finding is unexpected, as previous studies have associated being foreign-born with a higher likelihood of risky sexual behavior in general, except for sex under the influence [97]. This finding may be due to the minimal difference in acculturation levels between Latinas born in the United States and those born outside of the country. In our study, most Latina immigrants were likely to be exposed to traditional Latino cultural values and have families in which open discussion of sexual health and condom use is infrequent [20]. Additionally, most foreign-born participants have lived in the United States for more than two decades. 

Sixth, consistent with previous studies, our results indicate that increases in Latinas’ mental health symptoms were positively associated with risky sexual behaviors. Nonetheless, after controlling for all the significant variables, the effect of mental health symptoms was no longer significant; these findings are congruent with previous studies that reported a significant association between mental health and risky sexual behaviors—an association that should be further explored to determine which mental health disorders increase the risks of engaging in risky sexual behaviors [3]. Our results may support ongoing efforts to reduce substance misuse by addressing mental disorders among adult Latinas [98]. 

Seventh, not surprisingly, our findings indicate that older age is associated with a lower level of overall HIV risk behaviors and each of the three HIV risk behaviors, which is congruent with an extant body of research on the maturational effects of age on risky sexual behavior [99]. Our findings should be interpreted in light of several limitations. First, our sample is a community-based convenience non-clinical sample from Miami-Dade County. Although efforts were undertaken to include participants from major Latino subgroups, some groups (e.g., Latinas of Mexican and Puerto Rican origin) are underrepresented in Miami-Dade County; as such, the present sample is not representative of all Latinas in the United States. The present sample was representative of Latinas living in Miami-Dade County, but not of the larger U.S. Latino population; thus, future studies are needed with nationally representative samples to validate and enhance the generalizability of our results. Second, all data were collected via self-reported measures that are vulnerable to participant misrepresentation or error. Sexual behavior, particularly risky sexual behavior, is stigmatized among Latinas [100]. Although interviewers were trained to detect and address inconsistencies in participants’ responses, socially desirable responding may have occurred.

## 5. Conclusions

Our results indicate that being married, remarried, or with a main partner (compared with never married) is associated with multiple HIV risk behaviors. This result suggests that community HIV prevention intervention needs to continue targeting Latino women at the micro-level and Latinas’ personal relationships. Public health practitioners and policy makers should consider allocating funds and efforts to teach heterosexual Latino males the appropriate and consistent use of condoms and destigmatize condom use. Additionally, Latino women need continued education on condom use and safer sex negotiation skills. Involvement in the criminal justice system correlates with overall HIV risk behaviors, including sex under the influence, and sex without condoms. This result suggests that larger studies are needed to examine these variables in order to increase generalizability and health policy implications for Latinas from large groups, such as those of Mexican and Puerto Rican descent. Notwithstanding its limitations, the present study expands scientific knowledge on the social-cultural determinants and correlates of risky sexual behavior among adult Latinas. This study provides implications for the development of culturally appropriate preventions and interventions to address risky sexual behavior—based on the ecological systems theory; at the *meso* (community) level, parole programs should consider including mandatory HIV prevention education, similar to other court mandated training and courses, to increase parolees’ exposure to effective education on HIV prevention and thereby decrease the likelihood that these women will engage in risky sexual behaviors. Future research must measure more community-level risk factors and their influence on the correlates of risky sexual behavior of adult Latinas—findings from the present study provide the foundation for such investigations. The development of interventions tailored to adult Latinas and their main partners can lead to decreases in risky sexual behaviors and improved overall sexual health in this population. Bronfenbrenner defined the ecological theory as a theory that involves mutual interactions between humans and their environment. The fit between people and their environment influences whether the intervention’s results are successful or not. Parents and other family members have the longest history with the individual and play major roles in shaping patterns of development. In the current study, results suggest how women’s interaction with their partners and social environment influences their HIV risk and protective factors influencing their sexual behaviors.

Lastly, public health practitioners must consider risky sexual behavior prevention interventions with committed Latino heterosexual couples, as sex without condoms with the main partner is a primary form of infection for adult Latinas. Future policies and funding mechanisms must consider that male-to-female HIV infection is the primary mode of transmission and that future studies must fill the current gap in research and knowledge.

## Figures and Tables

**Table 1:** Descriptive analysis of the sample (*n* = 272).

Variable	Wave 1		Wave 2		Wave 3		Wave 4	
	*n*	%	*n*	%	*n*	%	*n*	%
Mother generation	135	50%	135	50%	135	50%	135	50%
U.S.-born	77	28%	77	28%	77	28%	77	28%
Mental Health								
Depression	85	31%	61	22%	52	19%	25	9%
**English-speaking level**								
Not at all	67	25%	56	21%	53	19%	55	20%
Fair	47	17%	57	21%	56	21%	45	17%
Good	75	28%	80	29%	67	25%	84	31%
Excellent	88	32%	85	31%	96	35%	88	32%
Intimate partner violence	106	39%	47	17%	32	12%	18	7%
Criminal involvement	38	14%	11	4%	3	1%	1	0%
**Marital Status**								
Married or remarried or with partner	73	27%	103	38%	110	40%	114	42%
Widowed or separated or divorced	107	39%	109	40%	105	39%	105	39%
Never married	92	34%	60	22%	57	21%	53	19%
Alcohol misuse	85	31%	56	21%	45	17%	52	19%
Drug misuse	34	13%	34	13%	35	13%	29	11%
HIV risk behaviors	191	70%	173	64%	165	61%	154	57%
Multiple sex partners	42	15%	35	13%	36	13%	23	8%
Sex under the influence	127	47%	115	42%	97	36%	90	33%
Sex without a condom	158	58%	149	55%	143	53%	136	50%
**Sociocultural**	**Mean**	**S.D.**	**Mean**	**S.D.**	**Mean**	**S.D.**	**Mean**	**S.D.**
Years in USA	19.50	13.88	25.26	13.92	26.31	13.96	27.43	13.92
Age at interview	39.88	15.92	39.88	15.92	39.88	15.92	39.88	15.92
Count of mental health symptoms	1.18	1.37	0.85	1.28	0.74	1.19	0.44	0.90
Tangible support	34.21	5.68	23.84	2.57	24.06	2.41	23.61	2.83
Belonging support	32.92	5.32	23.35	2.81	23.72	2.52	23.40	2.91
Relationship stress	12.83	4.76	11.10	4.12	10.75	4.07	9.90	3.40
Social stress	4.57	1.62	3.98	1.42	3.83	1.25	3.62	1.07
Neighborhood stress	7.04	1.98	6.86	2.05	6.84	2.03	6.61	1.80
Religious involvement	2.82	0.95	2.94	0.94	2.88	1.02	2.90	1.00
Attachment	91.98	17.73	92.83	19.49	94.13	20.36	95.69	19.79

**Table 2:** Bivariate analysis of the sociocultural determinants of risky sexual behaviors (*n* = 272).

Variable	HIV Risk Behaviors			Multiple Sex Partners			Sex under the Influence			Sex without a Condom		
	β	SE (β)	Exp. (β)	β	SE (β)	Exp. (β)	β	SE (β)	Exp. (β)	β	SE (β)	Exp. (β)
Years in USA	−0.02	0.01	0.99	−0.02	0.01	0.98	−0.01	0.01	0.99	0.00	0.01	1.00
Mother vs. daughter	−2.13 ***	0.23	0.12	−1.66 ***	0.28	0.19	−1.82 ***	0.21	0.16	−1.28 ***	0.23	0.28
Age at baseline	−0.09 ***	0.01	0.92	−0.07 ***	0.01	0.93	−0.08 ***	0.01	0.93	−0.05 ***	0.01	0.95
**Mental health**												
Depression	0.20	0.23	1.23	0.44	0.25	1.56	0.46 *	0.21	1.59	0.27	0.22	1.31
Total number of mental health symptoms	0.21 **	0.08	1.24	0.20 *	0.08	1.22	0.28 ***	0.07	1.32	0.20	0.08	1.23
**Sociocultural**												
U.S.-born	1.59 ***	0.32	4.90	0.76 **	0.27	2.14	1.65 ***	0.26	5.23	0.91 **	0.29	2.49
English speaking level	0.53 ***	0.11	1.71	0.28 **	0.11	1.32	0.55 ***	0.10	1.74	0.34 **	0.10	1.41
Tangible support	0.05 ***	0.02	1.06	0.02	0.02	1.02	0.04 **	0.01	1.04	0.02	0.01	1.02
Belonging support	0.07 ***	0.02	1.07	0.04 *	0.02	1.04	0.05 **	0.02	1.05	0.03	0.02	1.03
Intimate partner violence	1.08 ***	0.25	2.94	0.49 *	0.25	1.63	0.87 ***	0.21	2.39	0.98 ***	0.23	2.66
Relationship related stress	0.00	0.02	1.00	−0.01	0.03	0.99	0.01	0.02	1.01	0.01	0.02	1.01
Social stress	0.05	0.07	1.06	−0.04	0.08	0.96	0.01	0.06	1.01	0.07	0.06	1.07
Neighborhood stress	0.02	0.05	1.02	−0.01	0.06	0.99	0.02	0.05	1.02	0.03	0.05	1.03
Religious involvement	−0.27 *	0.11	0.77	−0.49 ***	0.12	0.61	−0.37 ***	0.10	0.69	−0.08	0.11	0.92
Criminal justice system involvement	2.82 ***	0.82	16.76	0.87 *	0.38	2.38	2.66 ***	0.51	14.28	1.68 ***	0.49	5.38
Attachment	−0.01	0.01	1.00	−0.02 **	0.01	0.98	0.00	0.01	1.00	0.00	0.01	1.00
**Marital Status**												1.00
Married or remarried or with partner	0.71 *	0.29	2.04	−1.91 ***	0.32	0.15	−0.66 **	0.24	0.52	1.34 ***	0.26	3.80
Widowed or separated or divorced	−1.65 ***	0.28	0.19	−1.02 ***	0.27	0.36	−1.55 ***	0.26	0.21	−0.92 ***	0.25	0.40
Never married	Ref			Ref			Ref			Ref		
Alcohol abuse	1.00 ***	0.27	2.70	0.96 ***	0.24	2.61	1.52 ***	0.22	4.56	0.68 **	0.24	1.97
Drug abuse	1.57 ***	0.37	4.80	1.07 ***	0.28	2.92	1.26 ***	0.28	3.52	1.37 ***	0.32	3.95

* *p* ≤ 0.05; ** *p* ≤ 0.01; *** *p* ≤ 0.001.

**Table 3:** Multivariate analysis of the sociocultural determinants of risky sexual behaviors (*n* = 272).

	HIV Risk Behaviors			Multiple Sex Partners			Sex under the Influence			Sex without a Condom		
	β	SE (β)	Exp. (β)	β	SE (β)	Exp. (β)	β	SE (β)	Exp. (β)	β	SE (β)	Exp. (β)
Years in USA	0.00	0.01	1.00	0.02	0.02	1.02	0.00	0.01	1.00	0.02	0.01	1.02
Mother vs. daughter	−0.49	0.42	0.61	−0.12	0.47	0.89	−0.22	0.39	0.80	−0.34	0.40	0.71
Age at baseline	−0.07 ***	0.02	0.93	−0.10 ***	0.02	0.90	−0.06 **	0.02	0.95	−0.05 **	0.02	0.96
**Mental health**												
Depression	NS			NS			−0.03	0.36	0.97	NS		
Total number of mental health symptoms	0.17	0.09	1.19	0.16	0.10	1.18	0.21	0.12	1.24	0.17	0.09	1.18
**Acculturation**												
U.S.-born	0.06	0.39	1.07	−0.51	0.38	0.60	0.35	0.34	1.42	−0.42	0.35	0.66
English speaking level	0.02	0.14	1.02	−0.24	0.16	0.78	0.08	0.13	1.08	−0.04	0.13	0.96
Tangible support	0.02	0.03	1.02	NS			0.02	0.03	1.02	NS		
Belonging support	0.04	0.03	1.04	0.01	0.02	1.01	0.00	0.03	1.00	NS		
Intimate partner violence	0.63 *	0.28	1.88	0.15	0.28	1.16	0.41	0.23	1.51	0.92 ***	0.25	2.51
Religious involvement	0.18	0.13	1.19	−0.19	0.14	0.83	0.05	0.12	1.05	NS		
Criminal justice system involvement	1.80 *	0.85	6.06	0.30	0.41	1.34	1.86 ***	0.52	6.43	1.10 *	0.49	3.01
Attachment	NS			−0.01	0.01	0.99	NS			NS		
**Marital Status**												
Married or remarried or with partner	1.95 ***	0.35	7.06	−1.32 ***	0.34	0.27	0.18	0.26	1.20	2.01 ***	0.29	7.47
Widowed or separated or divorced	−0.18	0.34	0.84	0.42	0.36	1.52	−0.40	0.31	0.67	−0.10	0.31	0.90
Never married	Ref			Ref			Ref			Ref		
Alcohol abuse	0.40	0.30	1.49	0.23	0.26	1.26	0.97 ***	0.24	2.64	0.29	0.25	1.34
Drug abuse	1.63 ***	0.42	5.09	0.74 *	0.32	2.11	0.87 **	0.30	2.40	1.26 **	0.33	3.53

* *p* ≤ 0.05; ** *p* ≤ 0.01; *** *p* ≤ 0.001. NS = not significant in bivariate analysis.
